# Regulating algorithmic tools in reproductive health: ethical and legal challenges

**DOI:** 10.3389/frph.2026.1771550

**Published:** 2026-05-08

**Authors:** Collins Chibueze Anokwuru, Moses Ifeatu Nwuzoh, Stanley Eneh, Ogechi Vinaprisca Ikhuoria, Gabriel Chidera Edeh, Onyeka Chukwudalu Ekwebene, Ephraim Ikpongifono Udokang, Francisca Onukansi, Gospel Chinaemerem Nwokocha, Samson Adiaetok Udoewah

**Affiliations:** 1 Corona Management Systems, Abuja, Nigeria; 2 Department of Public Health, Federal University of Technology, Owerri, Nigeria; 3 Youth in Research Hub, Enugu, Enugu, Nigeria; 4 Institute of Social Sciences, Sheffield Hallam University, Sheffield, United Kingdom; 5 Department of Community Health, Obafemi Awolowo University, Ile-Ife, Osun, Nigeria; 6 IVAN Research Institute, University of Nigeria, Nsukka, Enugu, Nigeria; 7 Department of Behavioral & Community Health, University of Maryland, College Park, MD, United States; 8 Department of Haematology/Blood Transfusion, Babcock University Teaching Hospital, Ilishan-Remo, Ogun State, Nigeria; 9 Department of Pediatrics, Ryan White Center for Pediatric Infectious Disease and Global Health, Indiana University School of Medicine, Indianapolis, IN, United States; 10 Faculty of Pharmacy, University of Uyo, Uyo, Akwa Ibom State, Nigeria; 11 Department of Plant Science and Biotechnology, University of Portharcourt, Port Harcourt, Nigeria; 12 Department of Public Health Nursing, Africa Centre of Excellence in Public Health and Toxicological Research, University of Port Harcourt, Port Harcourt, Nigeria

**Keywords:** accountability, algorithmic bias, artificial intelligence, data privacy, ethical challenges, ethical governance of AI, reproductive health

## Abstract

Artificial intelligence and algorithmic tools are increasingly integrated into reproductive healthcare, including fertility tracking applications, diagnostic systems, and clinical decision support tools. While these technologies may support improved access, personalisation, and decision making, their expansion is also associated with significant ethical and governance challenges in a domain shaped by legal risk, social norms, and deeply personal health decisions. These challenges include limited transparency, which may constrain informed consent and autonomy; risks of bias arising from unrepresentative data; heightened privacy concerns linked to sensitive reproductive information; and unclear accountability across developers, clinicians, and institutions. Importantly, these risks are not uniform, but are shaped by legal, social, and health system conditions, and may be particularly pronounced in low resource and legally restrictive settings. Despite growing attention to AI ethics, existing frameworks and guidance often remain high level and do not fully address how these challenges should be governed in context-sensitive reproductive health settings. This paper advances a governance-oriented perspective that integrates Healthcare 5.0 and reproductive justice frameworks to examine how these challenges emerge within adaptive socio-technical systems. It argues that existing governance approaches remain insufficiently responsive to structural and intersectional vulnerabilities and proposes a context aware approach that embeds human oversight, explainability, privacy protection, and accountability as core system level requirements. By positioning reproductive health as a critical test case for AI governance, this perspective highlights the need for enforceable, context-sensitive approaches that prioritise equity, autonomy, and the lived realities of affected populations.

## Introduction

Artificial intelligence (AI) and algorithmic tools are increasingly embedded in healthcare, reshaping diagnostics, clinical decision-making, and care delivery. AI can be understood as a subset of algorithmic tools, distinguished by its ability to learn from data and adapt over time, whereas algorithmic tools more broadly include rule-based and statistical systems operating on predefined logic (1, 2). In this paper, algorithmic tools refer to this broader category, while AI denotes adaptive, machine learning-based approaches. This growing integration is reflected in the expanding role of AI across clinical care, disease surveillance, and health system management, as highlighted by the World Health Organization (1). However, this rapid expansion has introduced significant ethical and legal challenges, including concerns related to opacity, privacy, bias, accountability, and the uneven distribution of risks and benefits (3).

Within this evolving landscape, reproductive health represents a particularly sensitive domain for algorithmic and AI applications. Areas such as fertility care, contraception, prenatal screening, and abortion involve decisions that are deeply personal and shaped by legal, social, and health system constraints (4). At the same time, digital tools such as fertility tracking applications routinely collect highly sensitive data on menstrual cycles, sexual activity, and reproductive intentions (5). More broadly, reproductive decision-making is influenced by contested norms, legal volatility, and unequal access to care. As a result, existing studies have identified risks related to privacy, autonomy, bias, and exclusion, particularly among marginalised populations, including adolescents, migrants, and individuals in low-resource or restrictive settings (6–8).

Despite these concerns, the literature on AI ethics remains largely generalised, with limited attention to reproductive health as a uniquely politicised and context-sensitive domain (9–12). Where reproductive health is addressed, analyses often examine ethical issues in isolation, with limited integration of structural inequality, intersectionality, and context-specific governance challenges (13–16). Similarly, existing WHO guidance, AI ethics frameworks, and SRHR-focused reviews provide important high-level principles (17–19), but offer limited direction on how governance should respond when algorithmic systems are deployed in settings characterised by legal restriction, stigma, and weak regulatory capacity.

In response to these gaps, this paper advances a governance-oriented perspective that integrates Healthcare 5.0 and reproductive justice through an intersectional lens, with a focus on legally restrictive and resource constrained contexts (20–23). Healthcare 5.0 conceptualises health systems as adaptive, human centric, and data driven socio-technical systems (20, 21), while reproductive justice foregrounds structural inequality, power, and uneven reproductive vulnerability (22).

Building on this, the paper advances three core claims: first, that ethical and legal risks associated with reproductive health AI are amplified in such settings; second, that existing governance approaches are insufficiently responsive to intersectional and structural vulnerabilities; and third, that integrating Healthcare 5.0 with reproductive justice provides a more appropriate framework for understanding reproductive health AI as an adaptive socio-technical system requiring continuous oversight, participatory governance, and context aware regulation.

To operationalise this perspective, the paper distinguishes between key classes of reproductive health algorithmic tools, including rule based applications, machine learning based diagnostic systems, decision support systems, and triage or prioritisation algorithms, recognising differences in autonomy, opacity, and associated risks (17, 18). These are analysed using the combined framework to examine how system design, context, and structural conditions shape governance needs. Together, this approach shifts the focus from describing ethical concerns to identifying governance priorities and regulatory conditions for responsible use.

### Applications of algorithmic tools in reproductive health

The use of algorithmic technologies in reproductive health diagnosis, counselling, and decision making is expanding across clinical and community settings (24, 25). These systems encompass different computational approaches with distinct functions and implications: rule based systems rely on predefined logic derived from expert knowledge, whereas machine learning models generate predictions by identifying patterns in large datasets (26, 27). Together, they underpin a growing ecosystem of tools, including tracking applications, diagnostic models, clinical decision support systems, and triage or prioritisation algorithms (5–8).

In practice, these technologies are increasingly embedded in care pathways. For example, rule based systems commonly underpin digital chatbots and virtual counselling platforms that provide guidance on contraception, sexual health, and family planning (25), while machine learning approaches are more often used in diagnostic and predictive tasks, such as AI assisted ultrasound tools for estimating gestational age or detecting abnormalities in prenatal care (28). However, many applications combine both approaches. Mobile fertility tracking tools integrate rule based logic with data driven features to generate personalised predictions (19, 29), and AI enabled decision support systems in prenatal diagnostics combine pattern recognition with clinical guidance (30). Emerging applications further include algorithmic support tools for abortion related information and management, as well as predictive models for adverse pregnancy outcomes such as pre eclampsia and postpartum haemorrhage (6, 31, 32).

Importantly, these tools raise distinct ethical and regulatory concerns. Rule based systems may oversimplify complex clinical scenarios and risk user misinterpretation (26), whereas machine learning systems raise issues of opacity, bias, and limited external validity, particularly when trained on non representative data (33, 34). Decision support and triage systems introduce additional governance challenges, as they may influence clinical judgement, shape care allocation, and affect access to time sensitive services, raising questions of accountability and fairness (32, 35). Distinguishing between these system types is therefore essential for determining appropriate oversight, validation, and accountability across contexts.

### Applications and governance challenges in low-resource and legally restrictive settings

In low resource and legally restrictive environments, algorithmic tools are increasingly used to address gaps in healthcare access, particularly where infrastructure, workforce capacity, and service availability are limited (36, 37). In such contexts, rule based systems are commonly deployed through mobile platforms, chatbots, and virtual counselling tools to provide guidance on contraception, sexual health, and family planning, especially where access to trained professionals is constrained (38). At the same time, machine learning applications, including AI assisted imaging and predictive models, are being introduced, although their deployment remains uneven due to limitations in infrastructure, data availability, and technical capacity (39). As a result, tools developed and validated in high income settings may not perform reliably in low resource environments, raising concerns about external validity and clinical safety (40, 41).

These applications illustrate how the context dependent dynamics outlined above operate in practice. The same algorithmic tool may produce different outcomes depending on legal, social, and health system conditions. For example, a fertility tracking application may support self management in settings with stronger data protection and access to care, but in legally restrictive environments it may also expose users to surveillance or legal risk where sensitive reproductive data are shared, insufficiently protected, or repurposed in ways that permit inference about pregnancy or abortion (42). Similarly, AI supported diagnostic tools may improve early detection in well resourced systems, yet in settings with limited follow up care they may generate uncertainty or decisions without adequate clinical support (43).

These risks are further shaped by structural constraints. In low resource settings, users may have limited capacity to question or contest algorithmic outputs, fewer alternative care pathways, and reduced legal or regulatory protection (44, 45). Consequently, concerns such as bias, privacy breaches, and over reliance on automated recommendations may be amplified by underlying inequalities and constrained health systems (17, 46).

From a governance perspective, this underscores the limits of applying frameworks developed in high income contexts without adaptation. Effective oversight therefore requires context-sensitive implementation, including local validation of tools, safeguards tailored to sensitive reproductive data, and enforceable mechanisms for accountability and user protection in practice (47, 48).

### Ethical issues and governance challenges in reproductive health AI: a Healthcare 5.0 perspective

The application of AI across reproductive health domains, including screening, diagnosis, decision support, and service delivery, is expanding rapidly and is often linked to progress towards universal access to sexual and reproductive healthcare and reproductive rights (24, 25, 38). However, the integration of algorithmic decision making into these sensitive areas introduces complex challenges relating to autonomy, equity, privacy, accountability, and legal risk (28–31). These challenges are particularly pronounced in reproductive health, where decision making is intimate, socially embedded, and shaped by restrictive laws, stigma, and unequal access to care.

Framing these challenges through the Healthcare 5.0 paradigm provides a basis for moving from descriptive ethics to governance focused analysis. Healthcare 5.0 emphasises human centricity, adaptability, ethical by design principles, and system level accountability within interconnected health ecosystems (20, 21, 23). In this paper, this perspective is integrated with a reproductive justice framework, which foregrounds structural inequality, power, and the conditions shaping reproductive autonomy and access to care (22). Together, these frameworks provide a complementary lens for understanding how algorithmic systems operate as socio-technical systems, in which data practices, model design, institutional use, and regulatory oversight interact to shape outcomes over time, particularly in constrained or legally restrictive contexts.

**Figure 1:**
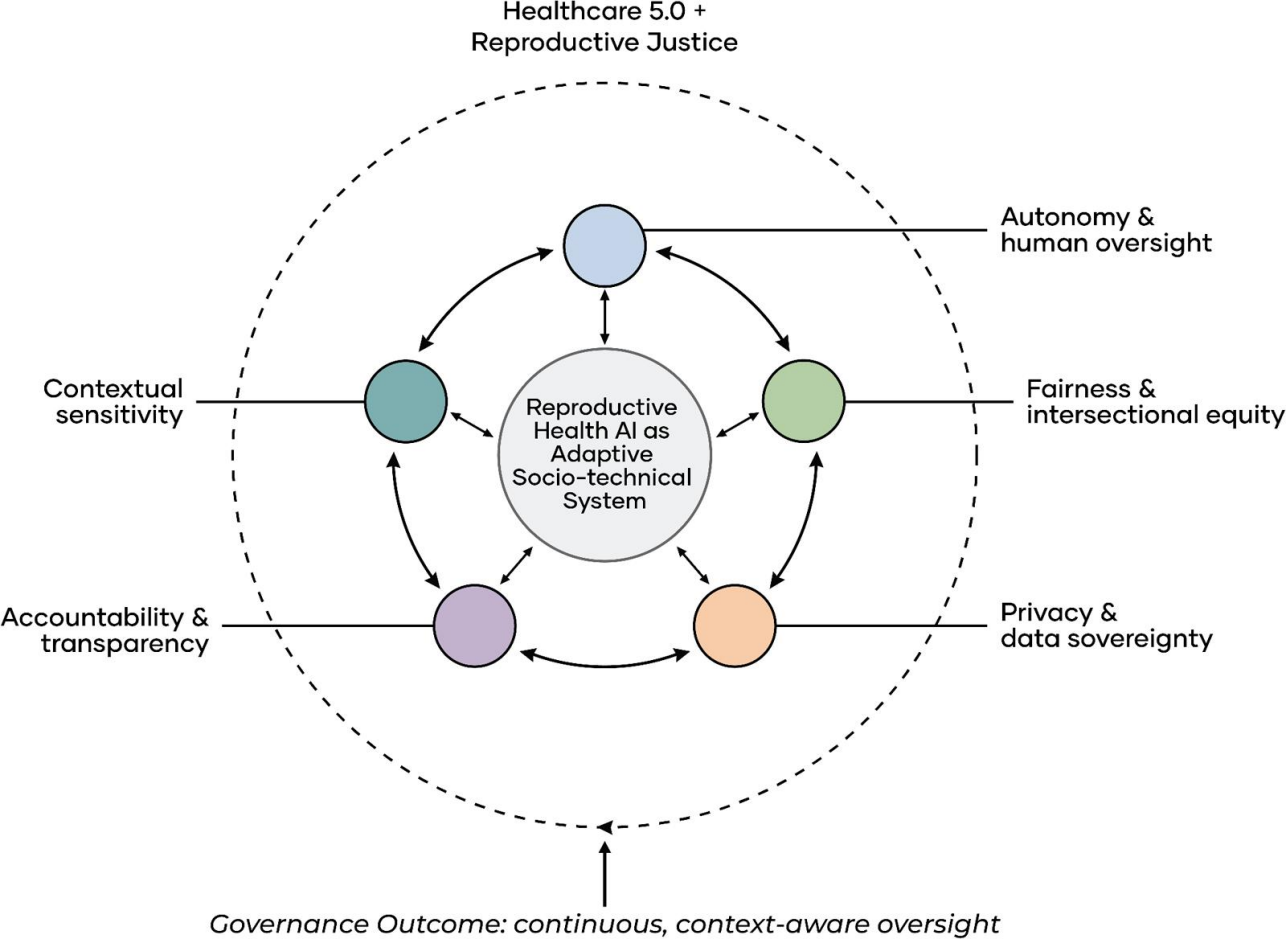
Integrated governance framework for reproductive health artificial intelligence combining Healthcare 5.0 and reproductive justice perspectives. This figure conceptualises reproductive health AI as an adaptive socio-technical system in which technological design, data practices, and regulatory oversight interact dynamically. It integrates Healthcare 5.0 principles, including human centricity, adaptability, and system level accountability, with reproductive justice dimensions that emphasise structural inequity, power, system-level accountability and context specific vulnerability. The outer dashed line is the enabling environment (Healthcare 5.0 and reproductive justice). The adaptive socio-technical system is indicated by the centre circle. The five surrounding nodes are interdependent governance domains, and these are in a radial layout to show that they are equally important and interact dynamically. The arrowheads between the nodes and the centre are two-way, indicating continuous feedback rather than a sequence. The five domains, which include autonomy and human oversight, fairness and intersectional equity, privacy and data sovereignty, accountability and transparency, and contextual sensitivity are not independent demands. Their interrelation implies that a failure in one area (e.g., privacy) can increase damage in another (e.g., fairness). The bottom label indicates the state of governance outcome: safe, equitable, and accountable use based on continuous, context-driven monitoring. This is particularly relevant because algorithms themselves are not risky but rather it is the interactions of the technical design, data practices, institutional use and regulatory oversight that create risks. It is especially important in environments where legal constraints and resource limitations are limiting, and where the gaps in the governance can magnify the damage. Accordingly, the framework supports a shift from one-time validation towards continuous, context aware governance, with implications for how regulators, developers, and deploying institutions design, evaluate, and oversee reproductive health AI systems. Adapted from (20–22).

Figure 1 presents this integrated framework as a conceptual synthesis for reproductive health AI governance. It illustrates how Healthcare 5.0 principles intersect with reproductive justice dimensions to define governance priorities, positioning autonomy, fairness, privacy, accountability, and contextual sensitivity as interdependent system level requirements rather than isolated ethical concerns (20–22). This perspective also highlights gaps in existing regulatory and institutional approaches, including limited explainability, weak bias auditing, insufficient protection of sensitive reproductive data, unclear accountability structures, and limited adaptation to local legal and sociocultural conditions. Accordingly, the sections that follow apply this integrated framework to examine how risks emerge, identify governance gaps, and clarify the oversight, design constraints, and institutional responsibilities required for equitable deployment of reproductive health AI.

### Autonomy and human oversight in reproductive health AI

From a Healthcare 5.0 perspective, autonomy in reproductive health AI is both a system level requirement and the capacity to make informed and independent decisions, ensuring that individuals retain meaningful control over their reproductive choices (20, 21, 49). From a reproductive justice perspective, this capacity is shaped by social, legal, and economic conditions that enable or constrain meaningful choice. However, the increasing use of algorithmic tools, including fertility tracking applications and AI supported clinical decision systems, introduces challenges to meaningful autonomy (45, 50).

These challenges vary across system types. Rule based tools may encourage reliance on outputs without understanding underlying assumptions, while machine learning systems may further limit informed decision making due to reduced transparency (37, 51). As a result, decision making may shift from active engagement to passive acceptance, particularly where outputs are presented as objective or clinically authoritative (52).

Current governance approaches often prioritise performance but do not ensure that users can question or contest system outputs (17, 53). This is especially concerning in reproductive health, where decisions carry legal and bodily consequences. These limitations are likely amplified in low resource or legally restrictive settings, where users may have limited capacity to challenge outputs and fewer alternative care options (44, 45).

From a governance perspective, autonomy requires more than transparency (17, 35). Systems must support interpretation, contestability, and human override, ensuring that final decision making remains with patients and clinicians. This need is reinforced by more adaptive systems, which increase the importance of clear limits on system autonomy and sustained human oversight.

### Bias, fairness, and intersectional inequities in reproductive health AI

Healthcare 5.0 emphasises adaptive and data driven systems that learn from diverse populations (20, 21). However, when algorithmic systems are developed using incomplete or unrepresentative data, they risk reproducing and amplifying existing inequities in reproductive healthcare (9, 10, 33). This is particularly relevant for machine learning models used in prediction and risk assessment, where performance depends on data quality, representativeness, and context (54–57).

Empirical evidence demonstrates how such biases translate into unequal outcomes. For example, risk prediction tools have been shown to underestimate illness burden among Black individuals compared with white individuals at similar predicted risk levels (33). Biases in training data have also contributed to the underrepresentation of people of colour in diagnostic systems, reflecting broader structural inequalities in data generation and access (33, 58). In reproductive health, these risks may be intensified when systems are applied in contexts that differ from those in which they were developed.

Current governance approaches often prioritise aggregate performance or simplified fairness metrics, which can obscure unequal impacts across population groups (17). However, reproductive health outcomes are shaped by intersecting factors including gender, socioeconomic status, legal status, migration, disability, and geography (23). As a result, fairness cannot be addressed through single axis comparisons alone, as such approaches risk obscuring compounded disadvantage and structural drivers of harm (23, 53). These risks are further amplified in low resource or legally restrictive settings, where data gaps and limited oversight intersect.

From a governance perspective, bias should therefore be understood as a system level issue rather than a technical limitation (20, 21). Addressing it requires representative data, intersectional evaluation, context specific validation, and continuous monitoring, supported by transdisciplinary input to ensure that algorithmic systems do not reinforce structural inequities in reproductive health.

### Privacy and data sovereignty in reproductive health AI

Within Healthcare 5.0, the integration of digital technologies requires careful governance of sensitive health data across interconnected systems (20, 21, 23). Reproductive health data are particularly sensitive, as they may reveal intimate information about menstrual cycles, fertility, sexual activity, pregnancy status, contraceptive use, and location (59). AI enabled tools, especially consumer facing applications such as fertility and menstrual tracking platforms, routinely collect such data, often with limited transparency regarding how they are stored, shared, or repurposed.

These data pose significant risks if misused or inadequately protected. In reproductive health contexts, such risks are shaped by legal and social environments. For example, in settings where abortion is restricted, use of tracking or related applications may expose individuals to surveillance or legal consequences if data are accessed or repurposed (60). Although data may be anonymised, re identification remains possible, particularly where datasets include location and behavioural metadata (61, 62). These risks are heightened in contexts where reproductive behaviours are criminalised or stigmatised.

From a reproductive justice perspective, these concerns extend beyond individual privacy to issues of power, control, and bodily autonomy (22). Data exposure may disproportionately affect individuals in restrictive environments or marginalised groups, whose reproductive choices are already subject to social or legal constraint (17). Existing governance approaches, which often rely on general data protection or anonymisation, do not adequately address these context specific risks or the sensitivity of reproductive data (22, 63).

From a governance perspective, privacy and data sovereignty must therefore be treated as core system level requirements rather than optional safeguards. This requires data minimisation, context-sensitive protections, and enforceable mechanisms that ensure meaningful user control over how reproductive data are collected, used, and shared across digital health systems.

### Accountability and transparency in reproductive health AI

Within Healthcare 5.0, AI systems are understood as components of broader socio-technical health ecosystems in which outcomes are shaped not only by algorithms, but also by data practices, system design, institutional use, and regulatory oversight (20, 21, 23). Accountability for reproductive health AI therefore extends beyond the model to developers, clinicians, deploying institutions, and regulators. This is particularly important given the potential clinical, emotional, and legal consequences of error or misinterpretation in this domain.

Transparency is widely recognised as a prerequisite for accountability in healthcare AI (64–67). However, in practice, many machine learning systems remain difficult to interpret even for clinicians and developers (17, 64, 68). This limits the ability to explain outputs, constrains patient understanding, and weakens opportunities to question or challenge decisions. In reproductive health, where decisions are often time sensitive and contested, insufficient transparency may contribute to delayed care or unrecognised error (31).

From a governance perspective, these limitations reflect deeper gaps in accountability frameworks, which often rely on *post hoc* transparency or limited disclosure. In this context, explainability should be treated as a design requirement linked to safe use and contestability rather than an optional feature (17, 34). At the same time, responsibility across the system lifecycle remains poorly defined, creating ambiguity over who is answerable when harm occurs (19, 54).

These challenges are intensified by more adaptive systems, where behaviour may change over time, making responsibility less clearly attributable to a single actor (17, 54, 66). Accordingly, accountability must be embedded through clear role allocation, continuous monitoring, and mechanisms that ensure responsibility remains traceable across all actors involved in system development and use (53).

### Contextual sensitivity

Contextual sensitivity refers to the need for AI systems to be designed and governed in ways that reflect local social, cultural, legal, and health system conditions (69). Global guidance emphasises that the risks and benefits of AI are not fixed, but are shaped by variation in regulatory frameworks, system capacity, and human rights protections (17, 70). In reproductive health, these factors are especially important, as restrictive laws, stigma, gender norms, and unequal digital access influence how technologies are used, trusted, and experienced, particularly in low income and Sub Saharan African settings (43, 71).

From a governance perspective, algorithmic risks should therefore be understood not as inherent to the technology, but as outcomes that emerge from the interaction between systems and their deployment environments (20, 23). A reproductive justice lens further highlights that these environments are structured by power, inequality, and uneven protection of reproductive rights, meaning that the same system may either reinforce or mitigate inequities depending on context (22, 23).

Accordingly, contextual sensitivity is a core governance requirement rather than a secondary consideration (46). It requires moving beyond one size fits all regulatory approaches towards models that incorporate legal conditions, sociocultural norms, and system capacity into design, validation, and oversight. In this sense, context defines what constitutes safe, equitable, and accountable use in practice (17, 46).

### Governance implications for reproductive health AI

The ethical and legal challenges discussed above highlight important gaps in current governance approaches and point to the need for more concrete, enforceable, and context aware oversight. Existing frameworks often emphasise high level principles such as fairness, transparency, and accountability, but provide limited guidance on how these should be operationalised in sensitive and context dependent domains such as reproductive health (11, 17, 34).

In practice, several limitations emerge. Autonomy may be constrained by opaque systems and the absence of consistent requirements for human oversight or explainability (12). Similarly, bias may persist where governance does not require representative data, routine auditing, or context specific validation, while fairness is often reduced to simplified demographic comparisons that overlook intersectional inequalities (23, 72). Privacy protections also remain insufficient when applied to highly sensitive reproductive data, particularly in settings where misuse or re identification may have serious social or legal consequences (73).

Accountability and transparency are frequently framed as principles rather than enforceable obligations, leaving responsibility unclear across developers, clinicians, institutions, and regulators (15). This challenge becomes more complex in adaptive systems, where system behaviour may change over time, making attribution of responsibility less straightforward (74). At the same time, many governance models do not adequately account for variation across legal, social, and health system contexts, which may shape how risks are experienced, particularly in resource constrained or legally restrictive settings (16, 75).

From a Healthcare 5.0 perspective, these limitations suggest the need to move towards system level governance that aligns design, deployment, and oversight. This includes enforceable requirements for human oversight, explainability, privacy protection, and intersectionality informed fairness, supported by clear accountability structures and context-sensitive implementation (17, 68). Such approaches should also incorporate transdisciplinary and participatory input to ensure that governance reflects the realities of those most affected. Table 1 summarises the key governance gaps and associated oversight measures, drawing on Healthcare 5.0 and reproductive justice perspectives, with particular attention to intersectionality, adaptive systems, and low-resource settings (20–22).

**Table 1:** Governance summary for reproductive health AI.

Ethical risk	Existing governance gap	Suggested oversight/governance measure
Autonomy, informed consent, and explainability	Existing frameworks do not consistently require meaningful human oversight, explainability, or contestability for high-stakes reproductive health AI (17, 19, 34, 53).	Mandate human oversight, explainability sufficient for informed and contestable use, and the ability for clinicians and users to question or override outputs (17, 19, 53).
Bias, fairness, and intersectional equity	Governance often lacks routine bias audits, representative datasets, and intersectionality-informed evaluation, relying instead on narrow fairness measures (16, 23, 33, 37, 57).	Require representative and context-relevant training data, routine bias audits, intersectional performance assessment, and monitoring for discriminatory impacts across subgroups (16, 23, 33, 57).
Privacy and reproductive data governance	General data governance frameworks are often insufficient for highly sensitive reproductive health data and may not adequately address misuse, surveillance, or re-identification risks (17, 42, 61, 63).	Implement privacy-first architectures, strong consent and access controls, limits on secondary use, and context-specific safeguards for sensitive reproductive data (17, 42, 63).
Accountability and adaptive system oversight	Responsibility is often unclear across developers, clinicians, institutions, and regulators, especially for adaptive systems that may change over time (19, 54, 64, 74).	Define clear responsibility across the AI lifecycle, require audit trails and transparency documentation, and introduce periodic review, post-deployment monitoring, and performance drift assessment (19, 54, 64, 74).
Local validation and contextual adaptation	Tools developed in high-income settings may be applied without sufficient adaptation to local legal, sociocultural, and health system conditions, especially in low-resource settings (39–41, 46, 48).	Require local and external validation, implementation testing in intended-use settings, and deployment rules that account for legal context, system capacity, and availability of follow-up care (39–41, 48).
Participation and reproductive justice	Governance approaches often insufficiently involve affected communities and may fail to address structural inequality, lived experience, and differential vulnerability (22, 23, 69, 76, 77).	Embed participatory governance by involving affected communities, clinicians, public health experts, ethicists, and reproductive justice stakeholders in design, evaluation, and oversight (22, 23, 76, 77).

## Conclusion

Reproductive healthcare is increasingly shaped by artificial intelligence and other algorithmic tools, with potential to improve access, accuracy, personalisation, and decision support. However, in a domain defined by legal risk, social norms, and deeply personal decision-making, these technologies also introduce significant ethical and governance challenges. Reproductive health should therefore be treated as a critical test case for responsible AI governance.

This perspective advances three claims. First, algorithmic risks are amplified in legally restrictive and resource constrained settings, where structural inequalities shape exposure to harm. Second, existing governance approaches remain insufficiently responsive to intersectional and structural vulnerabilities, often prioritising technical performance over social context. Third, integrating Healthcare 5.0 with reproductive justice provides a more appropriate basis for regulation by recognising reproductive health AI as an adaptive socio-technical system requiring continuous oversight.

To operationalise this, reproductive health AI systems should not be deployed unless minimum governance conditions are met. These include mandatory bias auditing and intersectional evaluation, context specific validation, explainability and meaningful human oversight for high risk use, privacy first data governance for sensitive reproductive data, and clearly defined accountability across the system lifecycle. Together, these shift governance from high level principles to enforceable obligations.

In practice, regulators must set and enforce standards; developers must design interpretable, privacy preserving, and equitable systems; and deploying institutions must ensure safe implementation, continuous monitoring, and human override. Responsibilities must remain clearly defined and enforceable as systems evolve.

Ultimately, the question is not whether AI should be used in reproductive health, but under what conditions. Ensuring safe, equitable, and accountable use requires governance that is enforceable, context aware, and grounded in lived realities. In line with Healthcare 5.0, this must operate at the level of adaptive socio-technical systems, requiring continuous oversight rather than one time approval.

## Data Availability

The original contributions presented in the study are included in the article/Supplementary Material, further inquiries can be directed to the corresponding author.
